# Insights, attitudes, and perceptions about asthma and its treatment: findings from a multinational survey of patients from Latin America

**DOI:** 10.1186/1939-4551-6-19

**Published:** 2013-11-04

**Authors:** Jorge F Maspero, Jose R Jardim, Alvaro Aranda, Paolo Tassinari C, Sandra N Gonzalez-Diaz, Raul H Sansores, Jorge J Moreno-Cantu, James E Fish

**Affiliations:** 1 Allergy and Respiratory Research Unit, Fundacion CIDEA, Paraguay 2035, Buenos Aires, 2 SS, Argentina; 2 Disciplina de Pneumologia, Universidade Federal de São Paulo, Rua Botucatú 740 - 3º andar, São Paulo, 04023-062, Brazil; 3 CardioPulmonary Research Group, Hospital Auxilio Mutuo, Apartado 191227, San Juan, PR 00918 1000, Puerto Rico; 4 Instituto de Inmunología, Universidad Central de Venezuela, Apartado postal 1050 - Ciudad Universitaria, Caracas, Distrito Capital 1051, Venezuela; 5 Centro Regional de Alergia e Inmunología Clínica, Hospital Universitario “Dr. José Eleuterio González”, Universidad Autónoma de Nuevo León, Ave. Madero y Gonzalitos s/n, Col. Mitras Centro, Monterrey, Nuevo León, 64460, Mexico; 6 Instituto Nacional de Enfermedades Respiratorias, Calzada de Tlalpan 4502, Belisario Domínguez Sección 16, Tlalpan, Mexico City, Distrito Federal 14080, Mexico; 7 Global Scientific and Medical Publications, Merck Sharp & Dohme Corp., 1 Merck Drive, Whitehouse Station, NJ 08889, USA; 8 Global Scientific Affairs, Merck Sharp & Dohme Corp., 1 Merck Drive, Whitehouse Station, NJ 08889, USA

**Keywords:** Asthma, Controlled, Guidelines, Exacerbation, Patient burden, Symptoms

## Abstract

**Background:**

In 2011 the Latin America Asthma Insight and Management (LA AIM) survey explored the realities of living with asthma. We investigated perception, knowledge, and attitudes related to asthma among Latin American asthma patients.

**Methods:**

Asthma patients aged ≥12 years from four Latin American countries (Argentina, Brazil, Mexico, Venezuela) and the Commonwealth of Puerto Rico responded to questions during face-to-face interviews. A sample size of 2,169 patients (approximately 400 patients/location) provided an accurate representation of asthma patients’ opinions. Questions probed respondents’ views on topics such as levels of asthma control, frequency and duration of exacerbations, and current and recent use of asthma medications.

**Results:**

A total of 2,169 adults or parents of children with asthma participated in the LA AIM survey. At least 20% of respondents experienced symptoms every day or night or most days or nights. Although 60% reported their disease as well or completely controlled, only 8% met guideline criteria for well-controlled asthma. 47% of respondents reported episodes when their asthma symptoms were more frequent or severe than normal, and 44% reported seeking acute care for asthma in the past year. Asthma patients in Latin America overestimated their degree of asthma control.

**Conclusions:**

The LA AIM survey demonstrated the discrepancy between patient perception of asthma control and guideline-mandated criteria. Additional education is required to teach patients that, by more closely following asthma management strategies outlined by current guidelines more patients can achieve adequate asthma control.

## Introduction

Asthma is a common disease across the world which affects people in all age groups and exerts a significant burden on patients and their families. Overall, asthma imposes a high societal burden in terms of productivity loss as well as resource utilization arising from poor asthma control. In recent years, educational initiatives were implemented to ensure that physicians know the latest evidence-based asthma treatment guidelines and are able to incorporate those guidelines in their clinical practices [1]. Evidence-based guidelines provide objective strategies to achieve and measure asthma control, although patients’ self-assessment of their asthma severity and level of control are not objective. Although guidelines offer an algorithm to assess disease control systematically and provide treatment recommendations that are easy to follow, patients worldwide misjudge their level of asthma control despite the availability of guidelines with clear-cut strategies. Over- or underestimating asthma control may result in ineffective treatment-related behaviors, and recent survey data from the United States suggest that patients may not be familiar with basic concepts in asthma management [2-4].

In 2011 the Latin America Asthma Insight and Management (LA AIM) survey was designed to ascertain the realities of living with asthma, the disconnect between expectations in asthma management and patient experience, and unmet needs. The LA AIM survey explored differences across Latin America in patient perception of asthma management practices and acceptable levels of asthma control. Using results from this survey we are able to evaluate the effectiveness of educational efforts to date. This is important because the effectiveness of any treatment strategy ultimately depends on actions taken by patients. Since patient perceptions drive treatment outcomes, it is crucial to provide patients with sufficient evidence to support informed decisions. The LA AIM survey explored asthma-related patient perceptions, behaviors, and presentation patterns, as well as recent trends in asthma management in Latin America.

## Methods

The LA AIM survey was designed to explore and document asthma-related patient perceptions, behaviors, and presentation patterns, as well as recent trends in asthma management. This survey was generally designed to follow the methods used in the AIM surveys in the US, Europe and Canada, and the Asia Pacific region. The LA AIM survey, developed by Abt SRBI (New York, NY, USA), was conducted in Argentina, Brazil, Mexico, Venezuela, and the Commonwealth of Puerto Rico to provide data representative of the Latin American population. Participants were randomly selected using national probability sampling. The study was not subject to formal ethics approval or formal consenting process in any country. The need for full consideration, and written informed consent, was waived by the Ethics Committee on Clinical Pharmacology at the CIDEA Foundation in Argentina, the Ethics Committee of the Universidade Federal de São Paulo in Brazil, the Ethics Committee of the Instituto Nacional Enfermedades Respiritorias Ismael Cosio Villegas in Mexico, the Cardio Pulmonary Research Center in Puerto Rico, and the Bioethics Committee for Research at Hospital de Clinicas Caracas in Venezuela.

The data were not collected by doctors or other health professionals. A survey organization based in each target country collected the data for this study (Table 1). Professional interviewers read a standard text to introduce the study and present the survey questions. For all countries and institutions, adequate procedures were in place to ensure the privacy and confidentiality of participants. Patient consent was explicitly obtained before the interview began, and the respondent could refuse to cooperate at any point in the interview. Identifiable personal information, including names, were neither collected nor maintained in the survey. The only unique identifier that existed at any time during the survey process was the telephone number in the sampling system during screening. That information was not provided with the rest of the survey data by the local data collection agency, and Abt SRBI received a totally de-identified data set. The study population consisted of adults age ≥18 years and parents of adolescents age 12–17 years with asthma based on physician-diagnosis and past-year medication use or asthma attacks. A total of 51,208 households were screened, yielding a sample of at least 400 patients in each country/area. Survey data were collected from each participant or their parent in a face-to-face interview and lasting no more than 35 minutes. Data were analyzed by individual country/area and for the entire study population. The survey consisted of 53 questions covering 5 asthma-related topics: 1) symptoms; 2) impact of asthma on daily life; 3) perceptions of asthma control; 4) exacerbations; and 5) treatment and medication.

**Table 1:** Demographics and sample characteristics

**Population adults/parents of adolescents (12–17 yrs) with asthma (physician diagnosed and past-year medication or asthma attacks)**		**Sampling frame screening of national sample of households**	**Interview length mean: 35 minutes**	**Patients**		
**Country**	**Survey organization**	**Households screened**	**Completed sample**	**Female, %**	**Mean age, yrs**	**Mean age at diagnosis, yrs**
Argentina	TNS-Gallup	16,321	436	66	40	19
Brazil	TNS	4,545	400	68	38	16
Mexico	Buendia Y Laredo	24,495	532	62	36	18
Puerto Rico	Interviewing Resources Corp.	2,193	401	64	43	19
Venezuela	StatMark	3,654	400	65	35	11
**LA AIM TOTAL**		51,208	2,169	67	37	15

AIM=Asthma insight and management.

Questions on symptoms covered the frequency of daytime and nighttime symptoms in the past 4 weeks, symptom frequency during the worst month over the past 12 months, and the frequency of sudden episodes of severe symptoms. To assess the impact of asthma on daily life, participants were asked about missed work or school days due to asthma, activity limitations, and overall productivity when asthma was at its worst. Regarding the perception of asthma control, participants were asked to rate their/their child’s level of asthma control over the past 4 weeks and the past year, and were asked to identify criteria for well-controlled asthma. These perceived levels of control were compared with asthma control classification based on GINA guidelines.^1^ Participants were also asked if they or their child had seen a doctor for exacerbations, symptom worsening, or sudden severe episodes over the past year. They were also asked if they or their child had to go to the hospital for an overnight stay or went to the emergency room for asthma, and how many times this occurred over the past 12 months.

Regarding asthma treatment and medication, participants were asked about use of asthma controller medication and quick relief or rescue medication, and if their doctor provided a written action plan for asthma management. Participants were also asked if they thought long term control medicines should be taken every day, if long term control medicines are not necessary when asthma symptoms are not experienced regularly, and if quick relief medicines can be used every day if needed.

All respondent data represent the percentage of patients who answered each respective survey question. No inferential analyses were conducted, and comparisons were for exploratory purposes only. For within-country comparisons in the LA AIM survey, the maximum margin of error for 400 individual country/region samples was ± 4.9% at the 95% confidence level. For across-country comparisons, when comparing 2 independent samples of 400, a difference of 6.9% is statistically significant at the 95% confidence level. Overall survey comparisons for 2000 survey samples had a maximum margin of error ±2.2% at the 95% confidence level.

## Results

Persons with asthma who were interviewed in the LA AIM survey were identified through the screening of 51,208 households, which found the household prevalence of asthma to be 3.4% in Argentina, 3.4% in Mexico, 8.8% in Brazil, 16.6% in Venezuela, and 18.3% in Puerto Rico. A total of 2,169 asthma patients were interviewed in the LA AIM survey, with 436 from Argentina, 400 from Brazil, 532 from Mexico, 401 from Puerto Rico and 400 from Venezuela. The mean age of respondents in the entire study population was 37 years, and the majority (67%) of respondents were female. In the entire study population, the mean age of respondents when their asthma was first diagnosed was 15 years, with median ages ranging from 7 years in Venezuela to 15 years in Argentina (Table 1).

### Asthma burden

The proportion of respondents who experienced asthma symptoms within the past 4 weeks was high. In the entire population, 24% experienced daytime symptoms every day or most days and 20% experienced nighttime symptoms every night or most nights (Table 2). Respondents reported shortness of breath during the day and awakening at night by shortness of breath as the most bothersome symptoms, in 24% and 22% of patients, respectively. Nearly half (47%) of the entire study population reported episodes in the past 12 months when asthma symptoms were more frequent or severe than normal (Table 2). The major triggers of asthma episodes in Latin America were dust exposure and weather changes. The proportion of respondents with asthma episodes was lowest in Mexico (42%), and was significantly higher in Argentina (57%) and Puerto Rico (61%) (Table 2). Slightly more than one-third of respondents (37%) reported that sudden severe episodes had a greater impact than day-to-day symptoms on their quality of life.

**Table 2:** Responses to selected survey questions

**Question**	**Latin America (n=2,169)**	**Argentina (n=436)**	**Brazil (n=400)**	**Mexico (n=532)**	**Puerto Rico (n=401)**	**Venezuela (n=400)**
Daytime symptom frequency, %						
Every day/most days	24	35	27	12	34	18
1-2 days/week	22	25	20	20	15	29
1-2 times/month	11	8	10	18	7	11
No daytime symptoms	44	32	44	50	43	42
Nighttime symptom frequency, %						
Every night/most nights	20	28	24	9	26	14
1-2 nights/week	16	20	16	16	15	18
1-2 times/month	10	8	11	12	5	10
No nighttime symptoms	53	45	50	62	53	58
Subjects with episodes in the past 12 months with asthma symptoms more frequent or more severe than normal, %	47	57	46	42	61	48
Median number of episodes	2	3	2	3	3	3
Subjects who agreed with the following statements, %						
Long-term control medicines should be taken every day	41	61	41	48	61	26
Long-term control medicines are not necessary when asthma symptoms are not experienced regularly	65	55	67	54	59	72
Quick relief medications can be used every day if needed	61	68	58	56	84	71
Subjects reporting ≥1 week of interrupted asthma controller medicine use, %						
<2 weeks	26	32	26	29	15	21
3-4 weeks	9	10	8	14	9	10
≥1 month	61	49	52	52	71	65
GINA 2009 asthma control levels						
Well controlled, %	8	5	9	9	8	3
Partly controlled, %	57	48	57	56	59	65
Uncontrolled, %	35	57	34	35	33	32

GINA=Global initiative for asthma.

**Figure 1:**
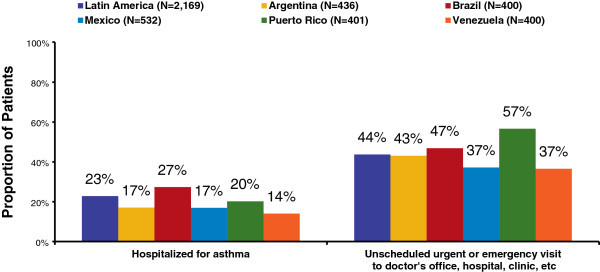
**Patient-reported acute treatment for asthma in past year question asked: (Have you/Has your child) been hospitalized for asthma in the past 12 months?.** Has (your/your child’s) asthma caused any unscheduled urgent or emergency visits to a doctor’s office, hospital, clinic, or somewhere else in the past 12 months?

The majority (59%) of respondents reported ever having an asthma episode in which they or their children had to go to the hospital for an overnight stay, and 11% reported ever having been admitted to an intensive care unit for an asthma episode. Survey questions also probed respondents’ views on the frequency and duration of exacerbations in the past year. Respondents were asked if they or their children went to the hospital for an overnight stay for asthma in the past 12 months, and if asthma caused any unscheduled urgent or emergency visits to a doctor’s office, hospital, clinic, or somewhere else in the past 12 months. In the entire population, 23% reported hospitalizations and 44% reported unscheduled urgent or emergency visits in the past 12 months (Figure 1). The proportion of respondents who went to the hospital for an overnight stay was significantly higher in Brazil (27%) than it was in the other LA countries/regions (14-20%). The proportion of unscheduled urgent or emergency visits was significantly higher in Brazil (47%) and Puerto Rico (57%) than it was in Mexico and Venezuela (37% each).

In the entire population, 40% of respondents reported that they missed work or school due to asthma over the past year, with a median of 3 days lost due to asthma. In addition, about half of respondents reported asthma-related activity limitations. The majority of respondents (70%) said asthma sometimes or often made them feel tired or fatigued, and 33% said asthma sometimes or often made them fearful. Overall, 39% of respondents thought their life was ever in danger during an asthma episode (Table 2).

### Controller medication use and perception of control

LA AIM survey respondents’ answers to questions about controller medication use show disparities in patients’ and/or doctor’s understanding of what it is proposed by the guidelines for recommended therapy and their attitudes about the use of control medication as well as quick relief medication. Overall, 42% of respondents reported that their doctor had developed a written action plan for asthma treatment, and 50% of these respondents (n=919) said their doctor reviewed the plan at every visit. Although 34% of respondents said they had heard of a peak flow meter, only 12% of these respondents actually had a peak flow meter. In the entire LA population, 41% of respondents agreed somewhat or strongly agreed with the statement that long-term control medication should be taken every day. However, 65% of patients felt that long-term use of asthma control medication was unnecessary when symptoms were not present and 61% overall felt that everyday use of rescue inhalers was acceptable (Table 2). Less than half (45%) of respondents reported use of controller medication over the past 4 weeks, whereas a majority (63%) of respondents reported use of quick relief medication over the past 4 weeks. In addition, 60% of respondents said they worry about using inhaled corticosteroids. The proportion of respondents reporting use of controller medication was highest in Puerto Rico (60%) and lowest in Venezuela (35%).

For methods of administering controller medication, 29% of respondents in the entire population reported the use of a pill, 20% reported the use of a nebulizer, and 55% reported the use of an inhaler (Table 2). Proportions of respondents reporting inhaler use for controller medication ranged from 42% in Brazil to 82% in Mexico. Among respondents who reported they or their child had stopped taking controller medication for a week or longer, 61% reported an interruption of ≥1 month (Table 2).

**Figure 2:**
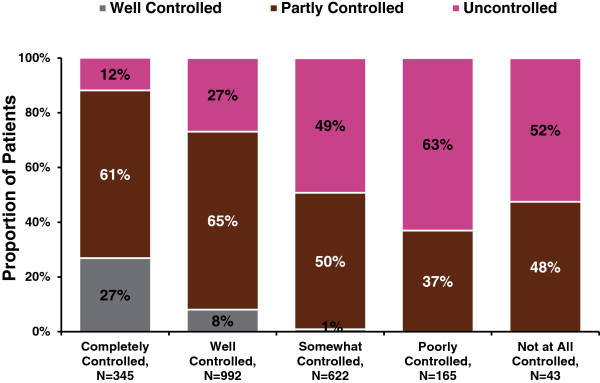
Asthma control classification by perceived control levels.

The survey found that asthma patients in Latin America overestimated their degree of asthma control. When respondents were asked how well they would say their or their child’s asthma has been controlled in the past 4 weeks, a majority (60%) of respondents reported their disease as completely controlled or well controlled. However, only 8% met the GINA guideline criteria for well-controlled asthma (Figure 2). Similar results were seen for respondents who perceived their asthma as being well controlled. Based on GINA control classification according to symptom frequency, activity limitation, and use of reliever treatment, 35% of LA AIM survey respondents would be considered to have uncontrolled asthma (Table 2).

In addition to overestimating their levels of asthma control, respondents had low expectations regarding the benefits of successful asthma management. About half of respondents (44-51%) would consider asthma to be well controlled if they or their child had only 2 urgent doctor visits, only 1 emergency room visit, or only 3 or 4 asthma attacks per year (Table 2).

## Discussion

Our findings from LA AIM reveal three relevant issues: 1) there is a high degree of agreement between countries in the Latin America region, although regional variability exists, with respect to the overall disease burden imposed by asthma, including asthma control, exacerbation rates, disease management approaches, and resource utilization; 2) asthma in LA, according to GINA, [1] is far from the standards of being well controlled; and 3) the burden of asthma identified in previous surveys of the region [5-9] continue to exist.

With a few exceptions the LA AIM found that patients with asthma from different countries responded with a very similar pattern of perceptions, behaviors and trends in asthma management. Most important is the fact that 56% and 47% of LA patients did report having daytime and nighttime symptoms, respectively, suggesting that they are used to living like this and misinterpret the concept of well controlled asthma. In this sense, although 60% of adults and adolescents with asthma in Latin America say they have been well or completely controlled in the past 4 weeks, only 8% would be classified as having controlled asthma according to GINA, whereas 57% would be classified as partly controlled, and 35% as uncontrolled. Similar results were observed in the US AIM study [4].

Almost half (47%) of adults and adolescents with asthma in Latin America reported episodes in the past year when their asthma symptoms are more frequent or severe than normal. Also, almost half (46%) of patients in Latin America report seeking acute care for asthma in the past 12 months, including being hospitalized for an overnight stay, emergency room visits, and other emergency medical care visits. Almost two-fifths of asthma patients in Latin America (39%) have felt their life was ever in danger during an acute episode. This may involve events such as an asthma attack followed by hospitalization for an overnight stay, admission to an intensive care unit, or loss of consciousness. Three out of five patients in Latin America express worry about using inhaled steroids (60%), as well as oral corticosteroids (61%). Fear of inhaled steroids directly promotes nonadherence to daily controller medication therapy, which can lead to episodes of asthma worsening with increased use of oral steroid bursts and emergency care or hospitalization for an overnight stay.

Patient expectations of asthma management are low; about half of patients consider their asthma well controlled if they have 2 urgent doctor visits per year, and about half of patients consider their asthma well controlled if they have 3 or 4 exacerbations per year. Reported patterns of medication use include infrequent utilization of controller therapy, interruptions in controller therapy, and excessive reliance on quick-relief agents. The acceptance of a certain degree of symptoms or exacerbations as normal obviously leads to overestimation of control status by patients. Many respondents did not have a clear understanding of adequate asthma control or how to measure control (e.g., use of a peak-flow meter). Overall, asthma imposes a high societal burden in terms of productivity loss as well as resource utilization arising from poor asthma control.

In 2003, the Asthma Insights and Reality in Latin America (AIRLA) survey assessed perception, knowledge, and attitudes related to asthma.^5^ Despite differences in their methodologies, the LA AIM and AIRLA surveys identified similar proportions of patients who experienced asthma symptoms over the past 4 weeks, and of patients who overestimated their asthma control based on GINA guidelines. This reveals ongoing discrepancies between patient perceptions of asthma control and guideline-defined control in LA surveys. Similar differences between patient perceptions of control and guideline-defined control were also observed in the US-AIM survey, [4] and also in the Asia-Pacific AIM survey conducted in 8 countries and Hong Kong [10].

## Conclusions

Based on patients’ self-reports of frequency and severity of symptoms and their perceptions of control, findings from this survey indicate that a large proportion of asthma patients in Latin America overestimate their levels of asthma control. The burden of asthma remains significantly high, and patients’ perception of control is not in agreement with actual level of asthma severity and symptoms. In spite of treatment guidelines, use of controller medication remains insufficient. It is important to interrupt patients’ acceptance of asthma burden, and recognize that persistent burden exists in spite of the availability of effective therapies. Actions are required to facilitate the assessment of asthma burden and effectiveness of asthma management, and to reverse patient-exhibited lack of conviction on treatment recommendations and suggested goals. A need exists in Latin America to implement educational efforts for physicians and patients on the management and control of asthma. Given that effective controller medications and guidelines for their proper use already exist, an important public health goal should be to promote their application in order to reduce the individual and societal burden of asthma.

## Competing interests

All authors received consultant fees from Merck Sharp & Dohme Corp., a subsidiary of Merck & Co., Inc., in conjunction with the development and execution of the Latin America AIM Survey.

## Authors’ contributions

All authors facilitated survey conduct in their respective countries, interpreted the survey findings, provided substantive suggestions for revision or critically reviewed subsequent iterations of the manuscript, and reviewed and approved the final version of the paper.
